# Self-Organizing and Routing Approach for Condition Monitoring of Railway Tunnels Based on Linear Wireless Sensor Network

**DOI:** 10.3390/s24206502

**Published:** 2024-10-10

**Authors:** Haibo Yang, Huidong Guo, Junying Jia, Zhengfeng Jia, Aiyang Ren

**Affiliations:** 1 College of Information Science and Engineering, Shenyang University of Technology, Shenyang 110167, China; guohd@smail.sut.edu.cn (H.G.); jiajunying@sut.edu.cn (J.J.); jiazhengfeng@smail.sut.edu.cn (Z.J.); renaiyang@smail.sut.edu.cn (A.R.); 2 Shenyang Key Laboratory of Advanced Computing and Application Innovation, Shenyang 110167, China

**Keywords:** thick LWSN, network self-organizing, network clustering, routing protocol, energy consumption

## Abstract

Real-time status monitoring is crucial in ensuring the safety of railway tunnel traffic. The primary monitoring method currently involves deploying sensors to form a Wireless Sensor Network (WSN). Due to the linear characteristics of railway tunnels, the resulting sensor networks usually have a linear topology known as a thick Linear Wireless Sensor Network (LWSN). In practice, sensors are deployed randomly within the area, and to balance the energy consumption among nodes and extend the network’s lifespan, this paper proposes a self-organizing network and routing method based on thick LWSNs. This method can discover the topology, form the network from randomly deployed sensor nodes, establish adjacency relationships, and automatically form clusters using a timing mechanism. In the routing, considering the cluster heads’ load, residual energy, and the distance to the sink node, the optimal next-hop cluster head is selected to minimize energy disparity among nodes. Simulation experiments demonstrate that this method has significant advantages in balancing network energy and extending network lifespan for LWSNs.

## 1. Introduction

With the development of the Internet of Things (IoT), WSNs have become a primary technology for monitoring railway tunnels [1,2,3]. WSNs consist of numerous sensors with sensing, computing, and communication capabilities [4,5,6,7,8]. During monitoring, nodes are randomly deployed within the railway tunnel and periodically send data to the base station. Since internal batteries supply the energy for sensors, nodes will fail once the battery is depleted [9]. Therefore, if energy consumption is imbalanced in the network, nodes may fail prematurely, leading to the network’s death [10].

Researchers have conducted extensive studies to address this issue. One approach involves clustering the network, where many nodes are divided into multiple clusters, with specific nodes acting as cluster heads. These cluster heads are responsible for forwarding data to the base station, reducing direct communication between nodes and the base station, lowering network energy consumption, and extending the network lifespan [11]. The key to clustering lies in how to elect cluster heads [12], and current methods mainly rely on factors such as the remaining energy of nodes and the distance to the base station for election [13,14]. Furthermore, researchers have designed routing protocols to identify the optimal paths for data transmission, aiming to reduce energy consumption [15,16,17].

In most cases, when monitoring railway tunnels, the nodes are arranged linearly, resulting in a linear topology known as an LWSN [18,19]. Compared with other types of WSNs, LWSNs exhibit fewer data transmission paths and lower network reliability due to their unique linear structure. Even the failure of a few nodes can lead to network disruption, especially in high-security railway tunnel monitoring scenarios. Therefore, traditional clustering and routing protocols based on WSNs are unsuitable for LWSNs. This paper proposes a self-organizing network and routing method based on an LWSN. After discovering the network topology, this method balances network energy consumption by optimizing network clustering and routing.

The main contributions of this paper are as follows:

1. Network topology discovery is achieved using a small number of anchor nodes. By the triangular relationship between two anchor nodes and an unknown node, the position of the unknown node can be uniquely determined. We also identify the minimum number of anchor nodes required, reducing packet redundancy and network costs;
2. Set different communication radii for nodes to achieve unequal clustering, reducing the reconstruction energy of clusters closer to the sink. Moreover, a timing mechanism is used to select the optimal cluster head;
3. In routing, cluster heads closer to the sink transmit data directly to the sink in a single hop, and cluster heads further away from the sink calculate a selection value based on the energy factor, distance factor, and load factor of candidate cluster heads. Based on the computed selection values, choose the next-hop cluster head for multihop transmission.

The remainder of this paper is organized as follows. Section 2 summarizes the related work. Section 3 introduces the system model of this study. Section 4 provides a detailed description of the implementation of the self-organizing method, including network topology discovery and clustering. Section 5 elaborates on the design details of the routing protocol. Section 6 compares the proposed method with the existing routing protocols. Section 7 concludes the paper.

## 2. Related Work

Recently, researchers have conducted extensive studies on using WSNs to monitor railway tunnels [20]. Since the nodes in the network are deployed randomly, network topology discovery before monitoring is necessary. In [21], a cloud-orchestrated physical topology discovery scheme for large-scale IoT systems is proposed. It comprises two stages, logical topology discovery and three-dimensional localization. The scheme improves the efficiency and accuracy of logical topology discovery using the parallel Metropolis–Hastings random walk algorithm. It employs the discovered logical topology and multidimensional scaling method combined with drones for global three-dimensional localization. However, despite the high precision of existing methods, they also involve high costs and complexity. In railway tunnel monitoring, the deployed nodes are often resource-constrained, and this scheme has high computation, communication, and energy consumption requirements. It may not be suitable for such resource-limited environments, especially with the need for cloud coordination and parallel computing.

Due to the often unattended or harsh environments of monitoring areas, extending the lifespan of WSNs presents a significant challenge, with energy balance being a key factor determining network lifespan. Extensive research has been conducted on this. In [22], a novel hybrid optimization algorithm, the Cuckoo Insisted–Rider Optimization Algorithm (CI-ROA) is proposed for cluster head selection in WSNs. The CI-ROA combines the advantages of the Rider Optimization Algorithm (ROA) and the Cuckoo Search Algorithm, aiming to significantly extend network lifespan by minimizing node distance, stabilizing energy, and reducing data transmission delay, but it may increase routing overhead. Although this strategy increases the algorithm’s complexity, it may also raise the difficulty of implementation and deployment, especially on resource-limited nodes. The algorithm’s performance is likely to be highly sensitive to the initial parameter settings. If the parameters are not chosen correctly, it may lead to suboptimal optimization results or even affect the network’s overall performance.

In [23], an improved Stable Election Protocol–Energy Consider (SEP-EC) is proposed for multilevel heterogeneous WSNs. Building on the original SEP, this version extends its applicability to multilevel heterogeneous networks and introduces optimization based on node residual energy to enhance energy balance. The SEP-EC combines multipath routing with load energy balancing and link prediction mechanisms, effectively balancing network energy, reducing packet loss and delay, and improving routing discovery efficiency. However, it may lead to more difficulty in algorithm implementation and maintenance. Additionally, it overly relies on the optimization of heterogeneity between nodes. If the energy levels of the nodes in the network are relatively similar, the protocol’s advantages may be challenging to realize fully. The paper in [24] proposes the Destination Oriented Routing Algorithm (DORA), which uses a mathematical analysis model to calculate the optimal transmission distance between any two nodes to minimize energy consumption and improve network efficiency. DORA selects the farthest forwarding node within the communication range based on this optimal distance. It constructs a multilink routing structure according to the direction toward the sink point, thereby effectively reducing energy loss. Moreover, the algorithm optimizes energy usage and extends network lifespan by employing a short-link strategy and dynamically adjusting the number of clusters and link configurations. However, DORA selects the farthest forwarding node within the communication range, which in some cases may increase the risk of packet transmission failure, especially when the communication environment between nodes is poor, or the channel quality is suboptimal, potentially leading to data loss or increased transmission delay. Although DORA optimizes energy usage by dynamically adjusting clusters and link configurations, these dynamic adjustments add to the algorithm’s execution overhead. Frequent adjustments, in particular, can increase communication and computation costs, impacting overall efficiency.

In [25], a balanced multipath routing and hybrid transmission approach is proposed. This method jointly optimizes multipath, multihop, and single-hop transmissions to design multiple nonoverlapping shortest paths, ensuring that the load is evenly distributed within hot-spot areas and effectively avoiding energy concentration. The algorithm first calculates the optimal transmission path from each node to the receiver and determines the best path selection and transmission periods to achieve balanced energy distribution. However, in practical applications, some nodes may still bear excessive load because they are located at the intersection of multiple shortest paths, leading to localized energy depletion and affecting the overall lifespan of the network.

In [26], an Improved Adaptive Ranking Energy-efficient Opportunistic Routing (I-AREOR) protocol is proposed to optimize energy efficiency in WSNs within Internet of Things (IoT) environments. The I-AREOR protocol selects optimal cluster heads by considering regional density, relative distance, and residual energy, effectively balancing energy consumption and maximizing network lifespan. The protocol adjusts energy parameters based on dynamic thresholds to extend the time until First Node Death (FND), Half Node Death (HND), and Last Node Death (LND). However, I-AREOR needs to dynamically adjust cluster head selection and energy parameters based on multiple factors (such as regional density, relative distance, and residual energy). This can increase computational overhead in resource-constrained sensor nodes, potentially affecting energy savings.

Selecting the optimal relay node is also crucial for balancing network energy. In [27], a relay selection algorithm for energy harvest WSNs is proposed. This algorithm optimizes the cooperation probability and uses an outage probability threshold as a constraint to control the relay nodes involved in cooperative transmission, thus conserving system energy. A multicriteria relay selection strategy is employed to choose the optimal relay node by considering the nodes’ solar energy status, network energy balance, signal-to-noise ratio, and outage probability. However, in environments with unstable lighting conditions or insufficient solar energy, the solar energy status of nodes may not reliably reflect their actual energy levels, thereby affecting the accuracy of relay selection.

In summary, railway tunnel monitoring faces challenges such as high algorithm complexity, significant communication and computation overhead, and high parameter sensitivity. Therefore, this paper proposes a self-organizing network and routing method specifically for railway tunnel monitoring to balance network energy and extend network lifespan:

1. Given that a large number of nodes are randomly deployed in railway tunnel monitoring and that there is significant overlap in communication ranges, the precision requirements for localization are lower. In the topology discovery, this paper uses a small number of anchor nodes. It locates unknown nodes through geometric relationships while introducing a method for calculating the minimum number of anchor nodes. This approach significantly reduces communication losses between nodes and lowers network deployment costs, thus improving deployment efficiency.
2. In the network clustering phase, this method builds on existing uneven clustering by applying a timing mechanism for cluster head selection. The timing duration is determined based on the node’s remaining energy, adjacency (degree), and position. This approach effectively balances energy consumption between clusters and results in smoother changes in the number of clusters, thereby achieving better network stability.
3. Considering the linear characteristics of the monitoring targets and the significant distance differences between nodes and the sink, this method employs a hybrid transmission approach combining single-hop and multihop transmissions to reduce routing overhead. Furthermore, the next hop cluster head in multihop transmissions is determined based on the cluster head’s energy, distance, and load factors, further balancing energy consumption among nodes.

Overall, the proposed method effectively reduces the difficulty and cost of network deployment. Through comparison with existing classical methods, the results demonstrate that the proposed method in this paper has approximately doubled the network’s energy efficiency, effectively balanced network energy, and extended the network’s lifespan.

## 3. System Model

### 3.1. Network Model

**Figure 1:**
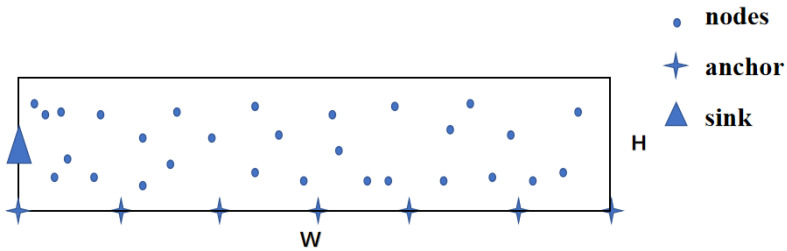
Long rectangular monitoring area.

The method proposed in this paper is specifically designed for the online monitoring of railway tunnels, and the network characteristics are as follows. The monitoring area is a W×H rectangular area (W>>H), with all sensor nodes used for data collection randomly deployed within this area. The total number of nodes is *n*, each with a unique identifier $N_{i}$, 0≤i≤(n−1). The sink is located at the center of the edge of the area and has sufficient energy. The monitoring area is illustrated in Figure 1.

We make the following assumptions regarding the sensor nodes:

1. All nodes remain stationary throughout the monitoring process.
2. The identifiers of the nodes are unique.
3. Nodes have the same initial energy and maximum communication radius.
4. The communication radius of the nodes can be adjusted based on the transmission power.

**Figure 2:**
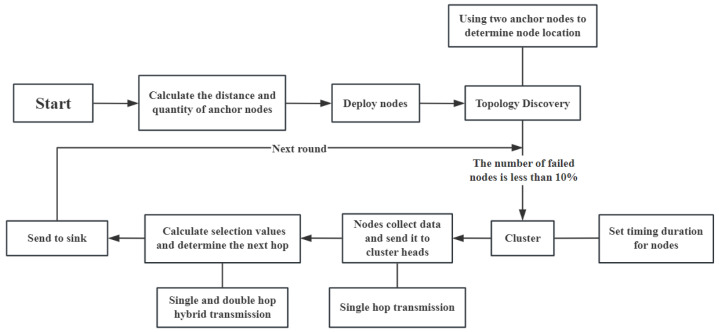
The workflow of thick LWSN.

In monitoring, the first step is determining the number of anchor nodes and their deployment spacing. Then, deploy anchor nodes and sensor nodes within the tunnel area. Following this, the self-organizing network process begins, including topology discovery and network clustering. In the routing phase, nodes collect data and send it to their cluster heads. The cluster heads will fuse the data and forward it to the sink through the routing protocol. Topology discovery is performed only once throughout this process, while clustering and routing phases occur periodically, with all active nodes collecting data in each round. Figure 2 illustrates the complete monitoring process.

### 3.2. Energy Consumption Model

During the operation of the nodes, energy consumption primarily comes from data reception and transmission. Therefore, based on the radio energy consumption model [28,29], the energy loss for a node transmitting a k−bit data packet is given by

(1) $$E_{T}\left(k,d\right)=\left\{\begin{array}{cc} kE_{elec}+k\varepsilon_{fs}\times d^{2}, & d<d_{0}\\ kE_{elec}+k\varepsilon_{amp}\times d^{4}, & d\geq d_{0} \end{array}\right.$$

Here, *k* represents the number of bytes in the transmitted data packet, and *d* denotes the data transmission distance. The distance threshold $d_{0}$ is given as

(2) $$d_{0}=\sqrt{\frac{\varepsilon_{fs}}{\varepsilon_{amp}}}$$

where $\varepsilon_{fs}$ and $\varepsilon_{amp}$ are energy consumption coefficients, and $E_{ele}$ is the radio energy coefficient. When receiving a k−bit data packet, the consumed energy is calculated as

(3) $$E_{R}\left(k\right)=kE_{elec}$$

## 4. Network Self-Organization

The goal of network self-organization is to achieve automatic configuration and management of the entire network through autonomous collaboration and coordination among nodes, thereby providing conditions for routing. This paper divides the network self-organization process into topology discovery and network clustering. In the topology discovery stage, a small number of anchor nodes are used, and the required number of anchor nodes is calculated to meet discovery needs while minimizing discovery complexity and reducing network costs. In the clustering stage, adjacency information is obtained through communication between nodes and used as one of the critical factors in electing cluster heads. Unequal clustering is implemented to balance network energy, and a timing mechanism is used to determine the order in which nodes become cluster heads.

### 4.1. Topology Discovery

**Figure 3:**
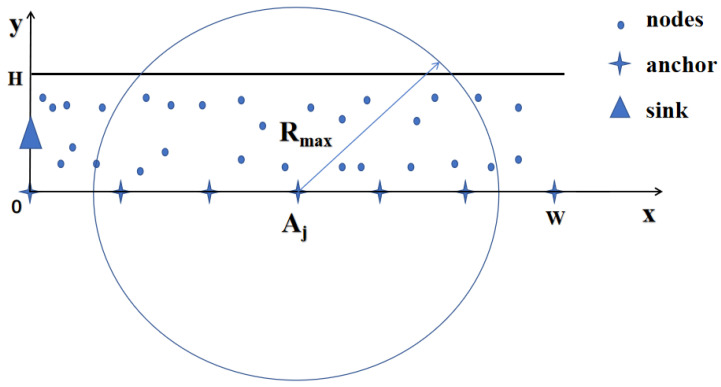
Monitoring area in Cartesian coordinate system.

**Figure 4:**
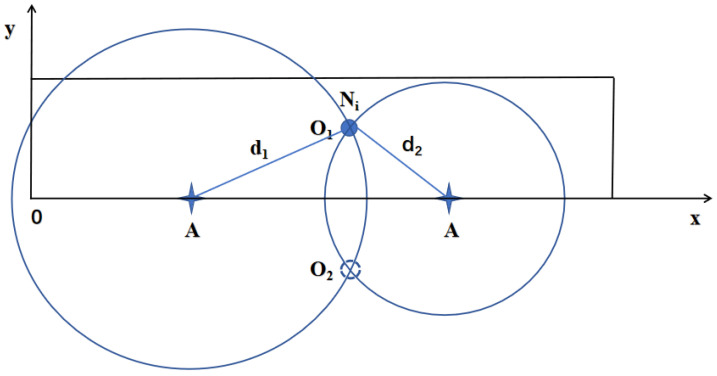
The unique location of node $N_{i}$.

The topology discovery of the network aims to determine the positions of all nodes within the network. We establish a Cartesian coordinate system with the area’s bottom edge as the *x*-axis and the left edge as the *y*-axis, as shown in Figure 3. It is well-known that determining the position of a point in a plane typically requires at least three known points. However, when anchor nodes are evenly distributed along the area’s bottom edge, only two anchor nodes are sufficient to determine a node’s position uniquely. As illustrated in Figure 4, if the distances from the unknown node $N_{i}$ to two anchor nodes are $d_{1}$ and $d_{2}$, respectively, the possible positions of node $N_{i}$ are at points $O_{1}$ and $O_{2}$. Since the anchor nodes are located along the area’s bottom edge, and the unknown node $N_{i}$ is within the area, the position of $N_{i}$ must be at point $O_{1}$.

**Figure 5:**
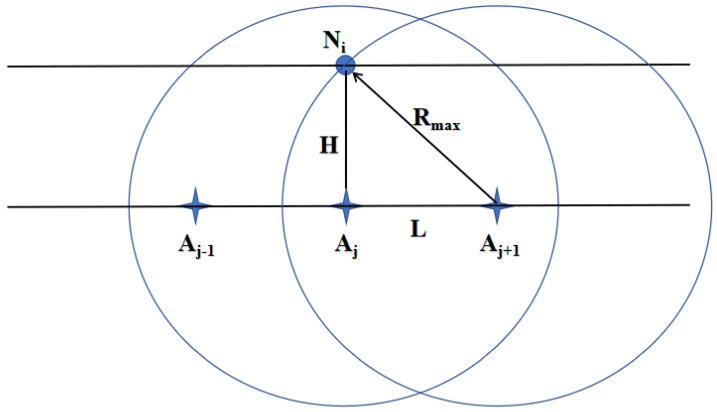
The maximum distance *L* between anchor nodes.

The distance between anchor nodes is *L*. When *L* is too large, some sensor nodes may fail to establish connections with at least two anchor nodes. When *L* is too small, the anchor nodes increase, leading to data redundancy and higher costs. To meet the self-organizing requirements and minimize the number of anchor nodes, *L* should be set reasonably. As shown in Figure 5, when sensor node $N_{i}$ is located above anchor node $A_{j}$, $N_{i}$ is farthest from $A_{j-1}$ and $A_{j+1}$. Therefore, *L* should satisfy the following formula:

(4) $$L=\lfloor \sqrt{R_{max}^{2}-H^{2}}\rfloor$$

Since the anchor nodes are evenly spaced, the following equation gives the number of anchor nodes *a*:

(5) $$a=\lceil \frac{W}{L}+1\rceil$$

During the topology discovery phase, all nodes will broadcast using their maximum communication radius, $R_{max}$. At least two anchor nodes will discover each node. The anchor nodes will determine their distance to the node using Formula (6), then reply with the distance $d_{ij}$ and their identifier $A_{j}$. When receiving data from anchor nodes, nodes will select the two nearest anchor nodes to determine their positions.

(6) $$d=10^{\frac{\left|\mathrm{RSSI}-A\right|}{10\times N}}$$

**Figure 6:**
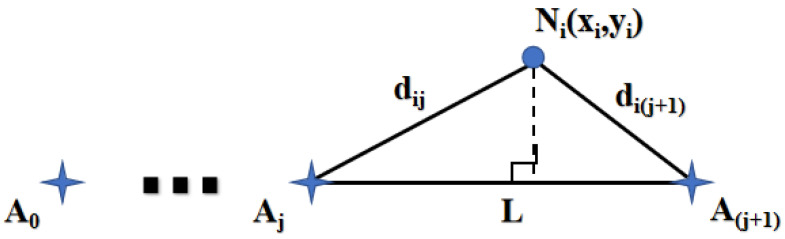
Coordinates of node $N_{i}$.

*A* represents the signal strength when the transmitter and receiver are one meter apart, and *N* is the environmental attenuation factor. Subsequently, the anchor nodes return the distance to the sensor node, which calculates its coordinates $\left(x_{i},y_{i}\right)$. The sensor node selects the two closest anchor nodes, as shown in Figure 6. Sensor node $N_{i}$ forms a triangle with the anchor nodes $A_{j}$ and $A_{j+1}$, and the area of this triangle can be calculated using Heron’s formula.    

(7) $$S=\sqrt{p\left(p-d_{ij}\right)\left(p-d_{i(j+1)}\right)\left(p-L\right)}$$

where *p* is the semiperimeter of the triangle, which is $\frac{d_{ij}+d_{i(j+1)}+L}{2}$. Since the length of the triangle base is *L*, we can determine the *y*-coordinate $y_{i}$ of $N_{i}$ according to the triangle area formula:    

(8) $$y_{i}=\frac{2S}{L}$$

and the following equation determines the *x*-coordinate $x_{i}$ of node $N_{i}$.

(9) $$x_{i}=j\times L+\sqrt{d_{ij}^{2}-y_{i}^{2}}$$

After determining their positions, the nodes send their coordinates and identifiers to the nearest anchor node, which forwards this information to the sink.

### 4.2. Establishment of Cluster

After the network topology discovery phase, nodes are set with different communication radii. Cluster heads near the sink undertake heavier forwarding tasks. Hence, they set smaller communication radii to reduce energy consumption during clustering. The following formula determines the node’s communication radius $R_{C}\left(N_{i}\right)$:

(10) $$R_{C}\left(N_{i}\right)=\left[1-c\frac{W-x_{i}}{W}\right]R_{max}$$

Here, the parameter *c* controls the communication radius range. The network enables unequal clustering by setting different communication radii for nodes.

**Figure 7:**
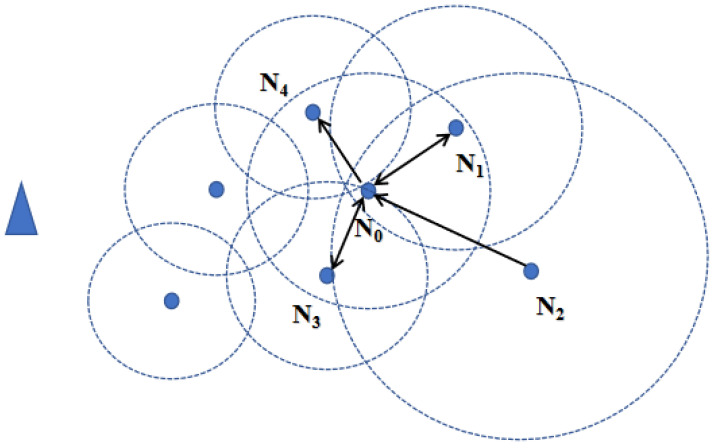
The connectivity of node $N_{0}$.

The selection of cluster heads is a crucial step in the clustering process, directly affecting the network’s energy consumption balance during subsequent work. At this stage, anchor nodes do not participate in clustering but serve as the medium for network coordination and management by the sink. We assume all sensor nodes have synchronized clocks. Before clustering begins, all nodes broadcast using their respective communication radii to discover neighboring nodes. The degree of a node is used to denote the number of neighboring nodes.

(11) $$N_{i}\cdot D=\left\{N_{j}|d\left(N_{i},N_{j}\right)\leq R_{C}\left(N_{i}\right)\right\}$$

where $d(N_{i},N_{j})$ represents the distance between nodes $N_{i}$ and $N_{j}$. When receiving broadcasts from other nodes, each node calculates the distances between them and determines whether they are neighbors based on their communication radius, updating its degree accordingly. As shown in Figure 7, node $N_{0}$ broadcasts within its communication range and receives broadcasts from other nodes. Because the communication radius increases with distance from the sink, $N_{0}$ only receives broadcasts from $N_{1}$, $N_{2}$, and $N_{3}$. Node $N_{0}$ then checks if the distances to these nodes are within its communication radius. From the figure, it can be observed that nodes $N_{1}$ and $N_{3}$ are neighbors of $N_{0}$.

We utilize a timing mechanism for clustering in the network, where each node has a timer whose duration $T(N_{i})$ is determined by Formula (12). At the start of clustering, all nodes activate their timers and enter a reception state. When the node receives the broadcast from another node, it joins that cluster if this node is its neighbor. If no broadcasts declaring a cluster head are received before the timer expires, the node broadcasts to become a cluster head. The timing formula is

(12) $$\begin{array}{c} T\left(N_{i}\right)=kT_{max}\left[1-\left(\alpha \frac{E_{R}\left(N_{i}\right)}{E_{0}}+\beta \frac{N_{i}\cdot D}{n}\right.\right.\left.\left.+\gamma (1-\frac{1}{d_{sink}\left(N_{i}\right)})\right)\right] \end{array}$$

where *k* is an adjustment factor ranging between (0.9,1), and $T_{max}$ is the maximum timing. $E_{R}\left(N_{i}\right)$ is the remaining energy of the node $N_{i}$, $d_{sink}\left(N_{i}\right)$ is the distance from the node $N_{i}$ to the sink, and α, β, and γ are coefficients for energy, degree, and position of the node, respectively, satisfying α+β+γ=1.

The formula shows that nodes with more remaining energy, higher degrees, and a farther distance from the sink are more likely to become cluster heads. The three coefficients determine how much these attributes influence a node’s becoming a cluster head. The cluster head election directly impacts the network’s energy balance in the current round. Therefore, in the subsequent experimental section, we obtain the optimal set of coefficients by comparison.

## 5. Energy Balanced Routing Protocol Based on Thick LWSN (EBRP-TL)

After each round of self-organization, ordinary nodes distributed across the monitoring area perceive the surrounding environment and transmit data to the base station for analysis, enabling real-time security monitoring of the entire monitoring area. During the intracluster routing phase, each node sends the data gathered to its respective cluster head via single-hop communication, and the cluster heads perform data fusion. We assume the packet size remains unchanged before and after data fusion is k−bit.

Due to the linear structure of the monitoring target, using a single-hop transmission approach for intercluster routing would result in significant energy consumption for cluster heads located far from the sink, demanding higher node power. Therefore, we employ a hybrid approach combining single-hop and multihop transmission methods. If the sink is within the communication radius of the cluster head, it directly transmits data to the sink via single-hop. Otherwise, the cluster head extends its communication radius three times to discover candidate cluster heads for the next hop. To ensure data forward transmission, the distance from candidate cluster heads to the sink must be less than that from the current cluster head node to the sink, as follows:

(13) $$NextCH\left(N_{i}\right)=\left\{N_{j}|d_{sink}\left(N_{j}\right)<d_{sink}\left(N_{i}\right)\right\}$$

All nodes have the same initial energy, but their energy consumption differs due to their varying positions and surrounding connectivity. When selecting the next-hop cluster head node, priority should be given to those with higher remaining energy to protect nodes with lower energy from premature depletion and failure and help balance the network’s energy consumption. Therefore, the remaining energy of the nodes is a crucial factor in selecting the next-hop node, and the energy factor is defined as follows:

(14) $$EF\left(N_{i}\right)=\frac{E_{R}\left(N_{i}\right)}{\bar{E}}$$

**Figure 8:**
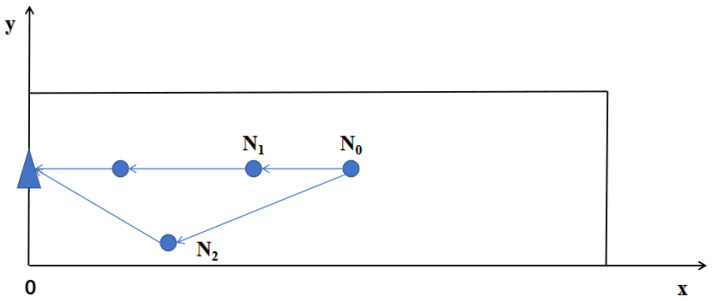
The connectivity of node $N_{i}$.

The transmission distance is an essential factor affecting node energy consumption. Under the same conditions, the farther the transmission distance, the more energy nodes consume. However, when monitoring linear infrastructure such as tunnels and bridges, where the length of the monitoring area is much greater than its width, as shown in Figure 8, nodes $N_{1}$ and $N_{2}$ are candidate cluster heads for node $N_{0}$. In this case, the transmission distances from $N_{0}$ through $N_{1}$ and node $N_{2}$ are almost equal. Therefore, $N_{0}$ prefers to select node $N_{2}$ as the next cluster head because this path involves fewer hops. Thus, those closer to the sink nodes are more likely to be chosen when selecting the next hop cluster head. The distance factor is defined as

(15) $$DF\left(N_{i}\right)=\frac{W-d_{sink}\left(N_{i}\right)}{d_{sink}\left(N_{i}\right)}$$

In intercluster routing, some cluster heads may be selected as next-hop nodes by multiple other cluster heads. This can lead to these nodes handling excessive data forwarding tasks in the current round, consuming too much energy and causing uneven energy distribution in the network. To prevent multiple nodes from choosing the same cluster head as the next hop, we define the load cluster heads in Formula (16).

(16) $$\begin{array}{c} PreCH\left(N_{i}\right)=\left\{N_{j}|d_{sink}\left(N_{j}\right)>d_{sink}\left(N_{i}\right)\right\} \end{array}$$

So, we choose cluster heads with fewer load cluster heads as the next hop nodes during the election process. The node’s load factor is as follows:

(17) $$LF\left(N_{i},N_{j}\right)=\frac{Pre_{max}\left(N_{i}\right)-PreCH\left(N_{j}\right)}{CH-Pre_{max}\left(N_{i}\right)}$$

Here, $Pre_{max}\left(N_{i}\right)$ represents the maximum number of the load cluster heads among candidate cluster heads of $N_{i}$. CH is the total number of cluster heads in the current round, calculated and communicated to all nodes by anchor nodes after clustering is completed.

We comprehensively consider the nodes’ energy, distance, and load factors, combining them into a selection value for choosing cluster heads. The cluster head $N_{i}$ will select the candidate cluster head $N_{j}$ with the highest selection value as the next-hop cluster head, and the definition of the selection value is as follows:

(18) $$select\left(N_{i},N_{j}\right)=EF\left(N_{j}\right)\times DF\left(N_{j}\right)\times LF\left(N_{i},N_{j}\right)$$

To elect the optimal next-hop cluster head, each cluster head node establishes a cluster head information table to calculate the selection value of candidate cluster heads and to inform other nodes about its information. Table 1 describes the relevant information on the cluster heads. Cluster heads broadcast the first four types of information in data packets and calculate the remaining information to add to their information table upon receiving packets from other cluster heads.

## 6. Test and Analysis of Results

We conducted simulation experiments using Python to verify the effectiveness of the proposed method. Additionally, we compare the routing protocol proposed with the classical routing protocols LEACH, E-LEACH, and EEUC under identical conditions. In our experiments, we randomly deploy 200 sensor nodes within a 600 m × 30 m monitoring area, with the sink node located at the center of the left edge of the region. To validate the robustness and reliability of the method, we set up two scenarios for the experiment: in Scene I, the sink is located at (0,15), and in Scene II, the sink is located at (650,15). The results in both scenarios are similar, so we use Scene I as an example for the explanation. Table 2 provides detailed parameter settings for the experiments.

**Figure 9:**
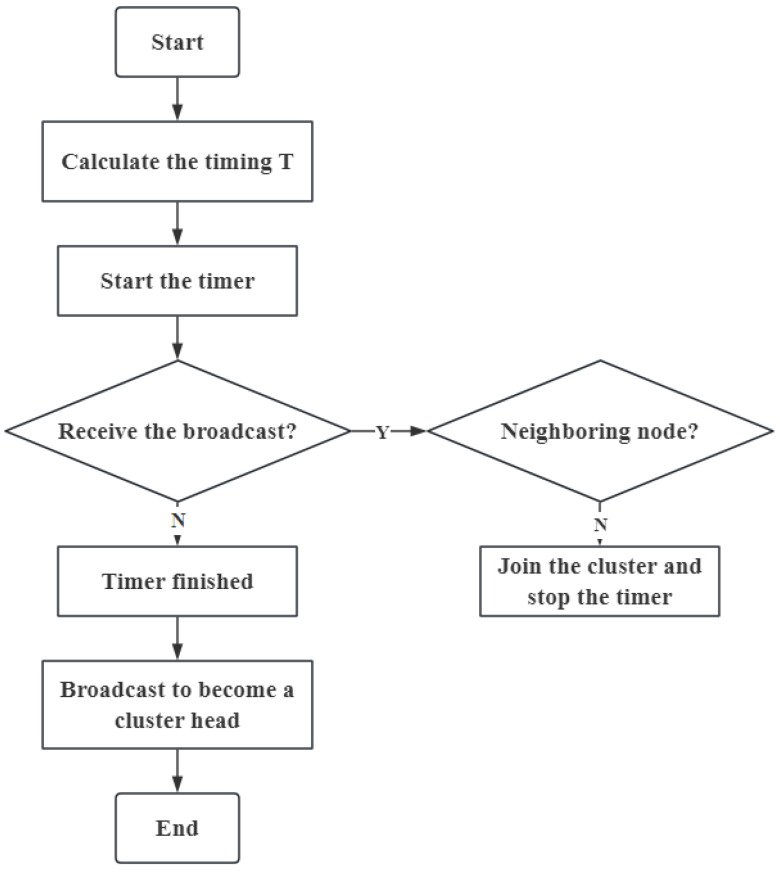
Clustering through the timing mechanism.

After the topology discovery phase, the network enters the clustering stage. Figure 9 presents the flowchart of nodes participating in the cluster head competition by using the timing Formula (12).

When the timer starts, nodes will enter the receiving state. If they receive a broadcast from other nodes becoming cluster heads before the timer ends, they will decide whether to join the cluster based on whether those nodes are their neighboring nodes. If no broadcasts are received by the end of the timer, the node will send out a broadcast to become a cluster head.

In the cluster head election process, we set each node’s timer duration $T(N_{i})$ according to Formula (12). In this equation, the timer duration $T(N_{i})$ is influenced by the node’s remaining energy, node degree, and the distance to the sink, where α, β, and γ represent their respective impact coefficients on $T(N_{i})$. The more residual energy the nodes have, the higher node degrees the nodes have, the greater the competition radius is, the shorter the set time is, it is more likely to become a cluster head. Timing began, and some nodes successively became cluster heads. In the early stages of the network’s operation, the energy levels between nodes are relatively balanced, and the timer duration is mainly determined by the distance from the node to the sink. After the network has been operating for a while, the nodes consume some energy, leading to an uneven energy distribution. At this point, the energy coefficient α is dominant. The impact of node degree is minimal, just in the clustering process, cluster heads with higher degrees can prevent the number of clusters in the network from becoming too large. Therefore, in the experiments, the values of α and γ are relatively large.

**Figure 10:**
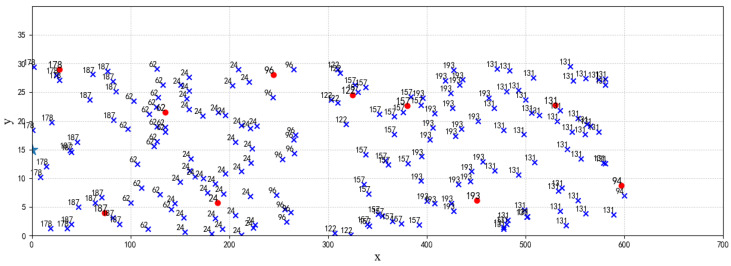
The clusters of network.

The cluster distribution in Figure 10 illustrates a round of network clustering. We unevenly distribute clusters by Formula (10), resulting in nodes farther from the sink having larger communication radii and thus forming larger clusters. For instance, node $CH_{131}$ is farther from the sink than $CH_{193}$, and its cluster covers a larger area. However, network clusters use the timing mechanism where cluster head election considers factors such as remaining node energy, node degree, and distance to the sink. Node $CH_{24}$ might have a shorter timer duration and become a cluster head earlier than node $CH_{96}$, resulting in surrounding nodes joining its cluster first. So it forms a smaller cluster despite node $CH_{96}$ being farther from the sink than node $CH_{24}$. This clustering approach reduces energy consumption within clusters closer to the sink, preserving energy for intercluster forwarding and balancing the network energy consumption.

After clustering is complete, the surviving nodes will begin monitoring. In each round of network operation, each node will send a monitoring data packet to its cluster head only once. In intracluster routing, nodes send data to the cluster head using a single-hop method, and the cluster head fuses the data. In intercluster routing, the cluster head decides on the transmission method based on the distance to the sink. The operational details of the cluster head in intercluster routing are provided in Algorithm 1. During intercluster routing, the communication radius of the cluster head is expanded to three times its original size. If the sink is within this communication radius, single-hop transmission occurs. Otherwise, the cluster head will collect information about its candidate cluster heads. The candidate cluster heads are closer to the sink, and the cluster head will select the best candidate cluster head as the next hop for multihop transmission based on Formula (18).
**Algorithm 1:** Intercluster Routing Algorithm
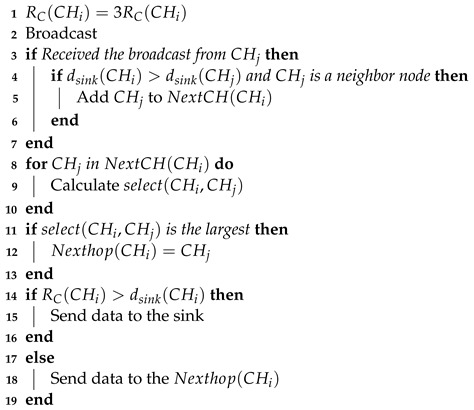


**Figure 11:**
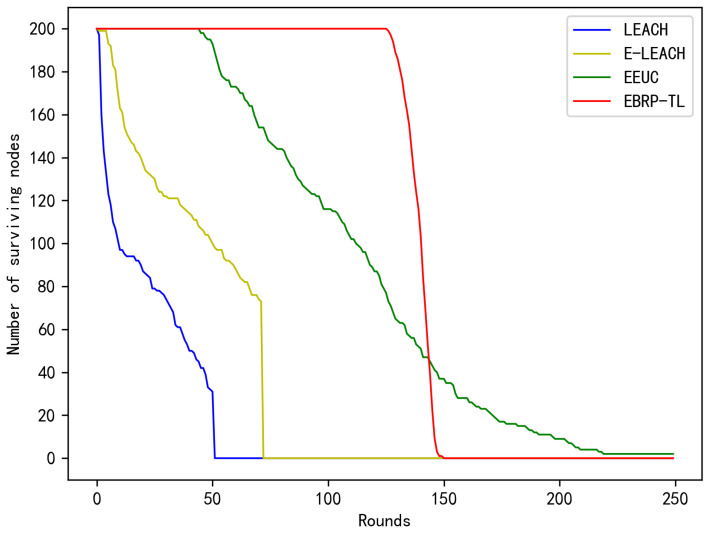
Number of surviving nodes.

When the energy consumption in a network becomes unbalanced, some nodes may prematurely exhaust their energy and fail. Therefore, to assess whether a network’s energy is balanced, one can evaluate the time the first node fails, reflecting the advantage of a routing protocol in balancing energy. The method proposed in this paper is suitable for the thick LWSNs in monitoring infrastructures such as railway tunnels and bridges. Hence, the number of failed nodes in the network had better be at most 10% of the total number of nodes for ensuring safety. Figure 11 compares the changes in total surviving nodes across different rounds for the LEACH, E-LEACH, EEUC, and EBRP-TL. With the LEACH protocol, the network experiences the first node failure early, and the death of the network is faster, primarily due to the randomness in cluster head election and the energy consumption from single-hop transmissions. E-LEACH improves upon LEACH by considering the node’s energy during cluster head election and reduces energy consumption during intercluster data transmission by introducing a root cluster head. As a result, it offers improvements compared with LEACH. The EEUC protocol conducts unequal clustering. Moreover, it utilizes a mix of single-hop and multihop transmissions, resulting in FND occurring only after approximately 50 rounds. In the EBRP-TL, cluster heads are elected based on node degree and distance to the sink. The protocol selects the optimal next-hop cluster head during intercluster routing. As a result, the protocol experiences its FND around 130 rounds, with only about 20 rounds passing between the first and last node failures. This demonstrates that the protocol achieves more balanced energy consumption among network nodes, extending the network’s lifespan.

**Figure 12:**
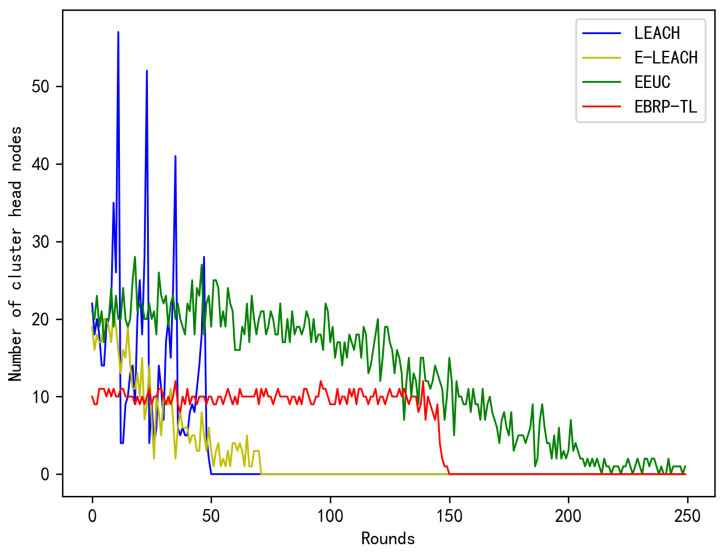
Number of cluster heads.

The number of clusters, or the number of cluster heads, is a crucial indicator for assessing network performance. Significant fluctuations in the number of cluster heads indicate substantial differences in energy consumption per round, leading to network imbalance and lower performance. The document in [30] proved that when the number of clusters reaches 3% to 5% of the total number of nodes, the network’s energy efficiency is at its highest. In this paper, the number of nodes is 200, so we assume that the network’s energy efficiency is highest when the number of cluster heads is at 5%. Figure 12 shows how the number of cluster heads varies with network rounds for three different protocols under the same conditions. In the LEACH protocol, where cluster heads are selected randomly, the number of cluster heads fluctuates considerably. Additionally, due to the rapid failure of nodes, the overall number of cluster heads shows a downward trend. E-LEACH takes the remaining energy of nodes into account during cluster head election, resulting in a significant reduction in the number of cluster heads. However, due to the inherent randomness in the cluster head election process, there is still considerable variation in the number of cluster heads. In contrast, while EEUC maintains a relatively stable number of cluster heads, this number is close to 10% of the total number of nodes, leading to lower energy efficiency. In contrast, EBRP-TL utilizes a timing mechanism for cluster head election based on uneven clustering, considering nodes’ remaining energy, degree, and distance to the sink. Nodes prioritized to become heads will have neighboring nodes within their communication radius join their cluster. This causes some nodes that might have become cluster heads to join clusters that broadcast earlier, electing the optimal cluster head and effectively controlling the number of clusters. As a result, the number of cluster heads corresponding to EBRP-TL in the graph remains around 5% of the total number of nodes, which is the optimal number of cluster heads.

**Figure 13:**
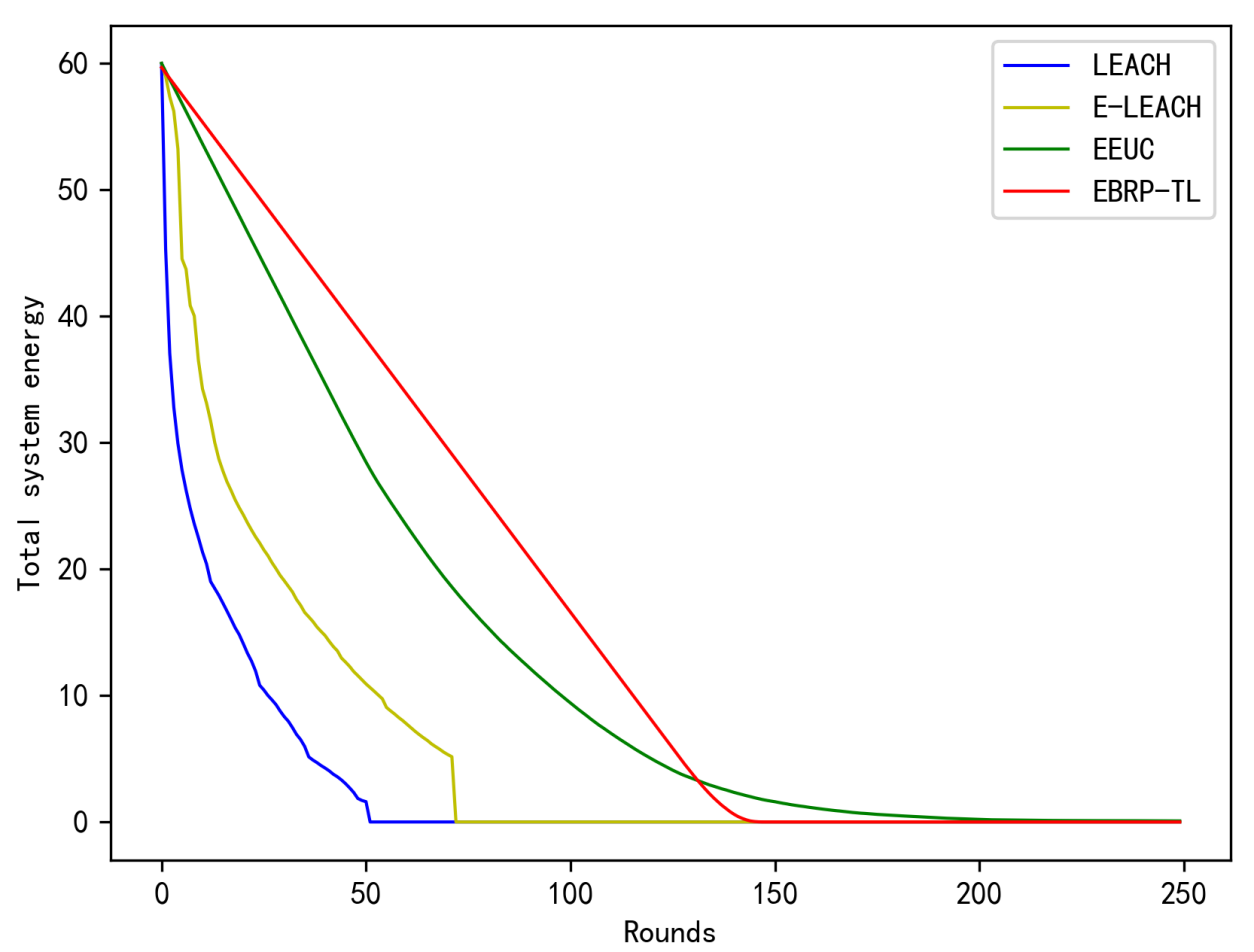
Total system energy.

To visually compare the energy balance differences among the three protocols, we analyzed how the total network energy changes with each round under different protocols. As shown in Figure 13, the initial total energy of the network is 60J, and the curve’s slope represents the energy change rate. Obviously, with the EBRP-TL, the network’s energy decreases almost uniformly, meaning the energy consumption per round is nearly equal. This result is consistent with the findings from the previous two experiments and further validates EBRP-TL’s advantage in energy balance.

The energy utilization rate refers to the ratio of energy consumed by the network during operation to the initial total energy. When the network experiences energy imbalance, the failure of a few nodes can disrupt regular network operation. However, the remaining nodes retain significant energy, leading to a decrease in energy utilization rate. After calculations, until the occurrence of 20 failed nodes (10% of total nodes), LEACH achieves an energy utilization rate of 38.2%, E-LEACH reaches 42.8%, and EEUC reaches 56.2%, whereas EBRP-TL reaches a high efficiency of 95.1%.

In summary, multiple experiments were conducted in different scenarios to compare the proposed method with existing classical protocols regarding FND, the number of cluster heads, and energy consumption. The results show that with EBRP-TL, the network experiences the latest FND, occurring after 130 rounds. The number of cluster heads consistently remains around 5% of the total nodes, and the total network energy decreases uniformly with each round. This result is expected because, during clustering, we not only adopted the existing unequal clustering method to reduce the reclustering energy of cluster head nodes closer to the sink but also introduced a timing mechanism. This mechanism determines the order in which nodes become cluster heads by Formula (12). Additionally, the timing mechanism ensures that the nodes prioritized as cluster heads absorb all neighboring nodes into their cluster, preventing the creation of closely spaced clusters, which would result in an excessive number of redundant clusters. During the routing phase, we selected the optimal next-hop cluster head for cluster heads far from the sink based on Formula (18), considering the cluster head’s energy, load, and location. This effectively protects low-energy, heavily loaded cluster heads and ensures the forward transmission of data.

## 7. Conclusions

For thick LWSNs used in railway tunnel monitoring, we propose a self-organizing network method and an energy-balanced routing protocol. In the network’s operation, anchor nodes play a role in node discovery and management. Regarding self-organization, we determine the positions of all nodes in the network by anchor node and establish adjacency relationships. Moreover, a timing mechanism is used to elect the optimal cluster heads to achieve unequal clustering. In the routing protocol, we select the next-hop cluster head node for intercluster data transmission based on various attributes of the cluster heads. Experimental results indicate that our method better balances network energy and extends lifespan compared with traditional energy-balanced routing protocols.

However, this study still has limitations. Firstly, using RSSI for distance calculation in the topology discovery process is susceptible to noise interference, which reduces the localization accuracy. Increasing the number of anchor nodes at the edges of the monitoring area or increasing the number of localization attempts may improve accuracy to some extent. In addition, future research plans to adopt the D2NN architecture proposed in the document in [31] for experiments, as it is more suitable for WSNs. Since this study is still in the simulation phase, issues such as hardware constraints and time synchronization are inevitable in practical applications, and addressing these will be one of the main focuses of our future research.

## Figures and Tables

**Table 1:** Cluster head information.

Mark	Describe
$CH_{i}$	ID of the cluster head *i*
$d_{sink}\left(CH_{i}\right)$	Distance from the cluster head to sink
$E_{R}\left(CH_{i}\right)$	Residual energy of the cluster head
$PreCH(CH_{i})$	Number of load cluster heads of the cluster head
$NextCH(CH_{i})$	Candidate cluster heads of the cluster head
$select(CH_{i},CH_{j})$	Selection value of candidate cluster head of the cluster head
$Nexthop(CH_{i})$	Next hop nodes of the cluster head

**Table 2:** Experimental parameter settings.

Parameters	Value
Node distribution (W×H)	600 m × 30 m
Sink node coordinates	(0, 15)/(650, 15)
The total number of network nodes *n*	200
Distance threshold $d_{0}$	87 m
Packet length	2000 bit
Packet length for control	100 bit
The maximum communication radius $R_{max}$	70 m
The initial energy of nodes $E_{0}$	0.3 J
Timing coefficient	0.5 + 0.1 + 0.4 = 1
Time adjustment factor *k*	0.95
Circuit consumes energy coefficient $E_{elec}$	5.0 × 10^−8^ J/bit
Data fusion energy Ech.C	5.0 × 10^−9^ J/bit
Energy coefficient of channel propagation model	$\varepsilon_{fs}$: 1.0 × 10^−11^ J/bit $\varepsilon_{amp}$: 1.3 × 10^−15^ J/bit

## Data Availability

Data are unavailable due to privacy.
